# A Novel High-Resolving Method for Genomic PCR-Fingerprinting of Enterobacteria

**Published:** 2010-04

**Authors:** A.S. Isaeva, E.E. Kulikov, K.K. Tarasyan, A.V. Letarov

**Affiliations:** Winogradsky Institute of Microbiology, Russian Academy of Sciences

**Keywords:** genomic fingerprinting, whole-cell PCR fingerprinting, insertion element, Enterobacterial diversity, strain differentiation

## Abstract

We developed a novel PCR–fingerprinting system for differentiation of enterobacterial strains using a single oligonucleotide primer IS1tr that matches the inverted terminal repeats of the IS1 insertion element. Compared to widely used BOX–PCR and ribotyping methods, our system features higher resolution allowing differentiation of closely related isolates that appear identical in BOX–PCR and ribotyping but differ in their phage sensitivity. The IS1–profiling system is less sensitive to the quality of the material and equipment used. At the same time, BOX–PCR is more universal and suitable for bacterial strain grouping and reconstruction of the low–distance phylogeny. Thus, our system represents an important supplement to the existing set of tools for bacterial strain differentiation; it is particularly valuable for a detailed investigation of highly divergent and rapidly evolving natural bacterial populations and for studies on coliphage ecology. However, some isolates could not be reliably differentiated by IS1–PCR, because of the low number of bands in their patterns. For improvement of IS1–fingerprinting characteristics, we offer to modify the system by introducing the second primer TR8834 hybridizing to the sequence of a transposase gene that is widely spread in enterobacterial genomes.

## INTRODUCTION

Animal (including human) bodies are ensembles of econiches populated by both various microorganisms and their viruses comprising the regular microflora. The animal (human) body is the main, if not sole, habitat for many microbial species [1]; however, atypical microorganisms may also be present [4]. The animal gut is one of the most densely populated parts of the body, and the host animal’s health is directly associated with the composition and state of its resident intestinal microflora [2]. In some cases, *Escherichia coli * and related enterobacteria, the most common mammalian intestinal colonists, cause migratory diseases in animals [2].

Existing methods of typing microorganisms that are based on phage sensitivity and antibiotic resistance tests are characterized by their inherent considerable drawbacks. In particular, phage typing is highly time–consuming and material–intensive, because it requires the creation and maintenance of phage libraries for typing enormous amounts of indigenous strains and, hence, is hardly appropriate for mass screening of isolates [5, 6, 11, 12, 16]. Antibiotic–resistant genes are often localized in plasmids that can be easily gained or lost in response to environmental changes, which raises the question of the stability of some “classical” phenotypic traits of different strains and of the dependence of resistance factors on environmental conditions [11].

Molecular differentiation of microbial strains is carried out today using universal DNA fingerprinting systems, such as ribotyping and repetitive element–PCR with primers corresponding to conserved repetitive (REP), extragenous (BOX), and intragenic (ERIC) elements of genomic DNA [11, 16]. BOX–fingerprinting with the primer BOXA1R complementary to the nucleotide sequence of boxA locus, as well as ERIC–PCR, is used for identifying sources of water pollution and for classification of * E. coli * isolates in wastewater and in horse, neat, and canine feces as well [5, 12]. The relatively high (70%) GC level in primers used for BOX– and ERIC–PCR [10, 11] allows them to hybridize and initiate DNA synthesis with partially complementary nucleotide templates at the annealing temperature used (52°C). This nonspecific annealing highly depends on temperature; so a slight deviation from the amplification parameters determined by the accuracy of the thermal cycler used can fundamentally influence the amplification results. Increasing the annealing temperature allows to achieve better accuracy, but in this case BOX– and ERIC–PCR lose their omnitude and require a specialized primer set for each bacterial genus [10]. This peculiarity complicates a comparison of the BOX and ERIC patterns obtained by different researchers in different series of experiments.

The ribotyping of *E. coli* indigenous strains is based on combining the strains into groups (ribotypes) sharing the homology of 16S rRNA gene sequences, the universal genome markers [4]. Several modifications of this method include systems with restriction enzyme profiling of the 16S rRNA gene PCR products or those with sequencing of the PCR products. The genes encoding rRNAs are highly conserved within any of the bacterial species, thus making virtually impossible intraspecific differentiation. The resolution power of this method is not enough for the tasks mentioned above, often cannot provide information on the taxonomic position of the studied microorganism below the specific rank, and is inconvenient in terms of outlay for analysis, number of stages, and interpretation of obtained data [5].

In this work, we intended to develop a reliable and easy–to–use universal molecular method for express–differentiation of enterobacteria on the basis of the PCR–amplification of their genomic DNA sequences and to test the method on isolates from natural animal gut microflora.

## MATERIALS AND METHODS

**Isolation of coliform strains**. Horse feces were sampled immediately after defecation into sterile plastic containers and stored at –70°C before use. Coliform bacteria were isolated as follows: a sample of 15–20 g wet weight was thawed at room temperature for 30 min and suspended in four volumes of physiological saline. Following shaking for 20 min at room temperature, the suspension dilutions 1:100 and 1:1,000 were seeded onto Petri dishes with LTA agar selective for enterobacteria: 20 g of Bacto–Triptose (Difco, USA), 5 g of lactose, 5 g of NaCl, 2.75 g of K_2_HPO_4_ (anhydrous), 2.75 g of KH_2_PO_4_ (anhydrous), 0.1 g of SDS, and distilled water up to 1,000 ml, pH 6.8.

The colonies grown on LTA agar (20 colonies from each of the three different samples) were streaked by sterile toothpicks onto the dishes with LB agar: 10 g of Tryptone (Amresco, Spain), 5 g of yeast extract (Difco, USA), 5 g of NaCl, 15 g of Bacto–Agar (Difco, USA), and distilled water up to 1,000 ml.

**Preparation of PCR templates**. A small portion of a single bacterial column was transferred with the bacteriological loop into a sterile Eppendorf tube containing 100 µl of deionized water, heated at +95°C for 20 min by using an Eppendorf Thermostat 5320 heating block, vortexed, and centrifuged for 1 min at 13,000 rpm on an Eppendorf 5414 benchtop microcentrifuge. Supernatant was used as template.

**IS1–fingerprinting**. We previously constructed the IS1tr primer (Golomidova * et al *., 2007): 5’–ATCAGTAAGTTGGA(G/A)(T/G)CATTACC–3’ that anneals to inverted terminal repeats of the insertion element IS1. The PCR reaction mixture (20 µL total volume) contained 67–mM Tris–HCl, pH 8.3, 17–mM (NH_4_)_2_SO_4_, 0.001% Tween–20, 2.5–mM MgCl_2_, 25–pM of the IS1tr primer, 0.2 mM of dNTP, 1.25 U of * Taq * –polymerase (Sigma), and 1 µL of the template under study. The reaction was conducted using either a Mini Personal Thermal Cycler (BIO–RAD) or previous generation cyclers Thercyc (DNA–Technology, Russia) and Perkin–Elmer Cetus (Perkin–Elmer).

The amplification protocol was as follows: denaturation for 30 s at 94°C; 30 cycles of denaturation for 15 s at 94°C, annealing for 30 s at 56°C, and elongation for 45 s at 72°C; and final elongation for 2 min at 72°C. The PCR products were analyzed by electrophoresis in 1% agarose gel.

We also constructed several other primers for improvement of strain differentiation (see the section “Results and Discussion”): IS2tr (5’–CAGATGTCTGGARATWYAGGGG–3’), IS3tr–L (5’–CCATATTACGTGGGTAGGATCA–3’), IS3tr–R (5’–CCACTATTGCTGGGTAAGATCA–3’), IS4tr (5’–TSCTTAACTGACTGGCATTA–3’), IS5tr (5’–SSRCTTRTTCGCACCTTCC–3’), IS30tr (5’–TGTTGCRTTGACMRATTGAATCTACA–3’), TR8D (5’–ATGCACGTCATACTCTTTTTT–3’), TR8R (5’–AAGAGTATGACGTGCATCCTA–3’), and TR8834 (5’–ATCGGCGATGCGTTGACGAAT–3’).

**Rep–PCR**. BOX–fingerprinting was carried out according to the authors’ protocol [15]. The BOX primer A1R (5’–CTACGGCAAGGCGACGCTGACG–3’) was used instead of the IS1tr primer in the reaction mixture above. However, the amplification protocol was essentially different. The reaction began with denaturation for 2 min at 95°C, followed by 30 cycles of denaturation for 3 s at 94°C and 30 s at 92°C, annealing for 1 min at 50°C, and elongation for 8 min at 65°C; and the final elongation for 8 min at 65°C. The overall program took about seven hours. PCR products were detected according to the standard protocol (see above).

**Ribotyping of *E. coli* autostrains**. The genes encoding 16S rRNA were amplified using the primers 27F (5’–AGAGTTGATCMTGGCTCAG–3’) and 1492R (5’–GGTTACCTTGTTACGACTT–3’) [4] that are universal for eubacteria. Endonuclease restriction profiling was fulfilled using *Hind * III and * Hae * III restrictases (Fermentas, Lithuania). The restriction products were analyzed by electrophoresis in 2% agarose gel.

Phage sensitivity of coliform isolates was estimated according to the Gratia bilayer method on a LB medium. The upper layer was LB containing 0.6% Bacto–Agar.

## RESULTS AND DISCUSSION

Since we aimed to develop a robust and convenient PCR–system for high–resolution genome typing of coliform strains, field testing of the novel system was necessary on a series of natural coliform isolates. So, the indigenous enterobacteria isolated from the feces of three horses served as the subject of the inquiry. Eighty various clones were chosen from the colonies grown on a LTA medium selective for enterobacteria.

**IS1–fingerprinting system**. We have developed a new system for genomic PCR–fingerprinting [6]. The PCR template is a crude DNA extract from heated cells rather than the purified DNA. The reaction uses single oligonucleotide primer annealing with inverted terminal repeats of the insertion element IS1 that is widely distributed in enterobacterial genomes [3], so that the primer 3’–end is directed. outwards of the element. Thus, the sequences amplified are those that are localized between either IS1 copies or other hybridization sites which are not associated with IS1 copies but may represent the remaiders of lost insertion elements. The length of specific reaction products depends on the relative position of IS elements or other hybridization sites in the bacterial chromosome, but it does not exceed the limit defined by the PCR conditions. The reaction products may be separated and analyzed by routine DNA electrophoresis in agarose gel [6].

**Fig. 1:**
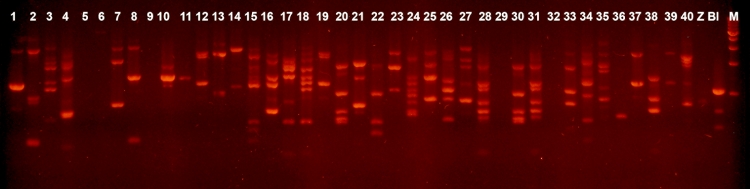
IS1-fingerprinting of indigenous coliform strains. Lanes 1-20 – strains isolated from sample of horse feces No 1 Lanes 21-22 – strains isolated from sample of horse feces No 2 Z-: *E.coli Z85*; Bl – *E.coli BL21* Marker – 1kb DNA ladder (Fermentas)

The data of PCR with the IS1 primer show a distinct pattern of the reaction products for each coliform strain. All bands are well–separated in agarose gel. In most cases, their number varies from two to ten, thus simplifying evaluation of identical or closely related IS1–patterns. For instance, two identical patterns (Fig.1, lanes 12 and 19) were found among the indigenous strains isolated from the first fecal sample, and two pairs of identical patterns (Fig. 1, lanes 28, 31 and 37, 39) – among those isolated from the second one. Besides, two identical patterns were found between the strains from the first and second samples (Fig. 1, lanes 20 and 30).

**Reproducibility and sensitivity of IS1–fingerprinting**. The test for resistance of genomic DNA template amplification to various physical and chemical factors has shown that heating the template for 10 min at 100°C has no effect on IS1–fingerprinting, as compared to control; thus, possible deviations from heating parameters during template preparation would not influence the results. It is notable that in the course of this work (about three months), the templates were stored in a freezer and repeatedly underwent thawing and freezing without any effect on both the quality and quantity of the IS1–PCR products (and coliform IS1–fingerprinting patterns as well). It is worth noting, however, that the excess of heat–lyzed biomass in the reaction mixture can inhibit PCR, so positive control is necessary in each template series, with the use of the strain certainly providing a specific pattern.

**Fig. 2:**
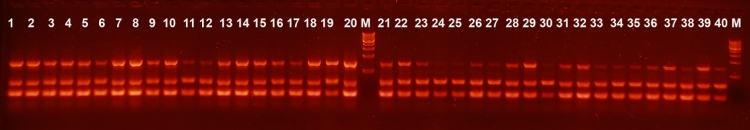
Test for stability of IS1-pattern through passages of the strain in the liquid medium. No difference between subclones of initial strain (lanes 1-20) and its subclones obtained after 5 passages in liquid medium (lanes 21-40) was observed. Marker – 1kb DNA ladder (Fermentas)

To check for the stability of IS1–fingerprinting through generations, we chose a strain with an easy–to–read IS1 pattern. Then, the strain was passed through five sequential passages in a liquid LB medium. The culture dilutions from the first and last passages were plated on LB agar for single colonies isolation. The IS1–PCR of randomized 20 colonies randomly chosen from each passage showed no deviation of subclone patterns from the initial one (Fig. 2). Some difference in the intensities of individual DNA bands after electrophoresis in agarose gel might result from the nonstandardized amount of the DNA template in the PCR mixture. The indistinguishable genomic patterns of the initial strain and its offspring at a limited number (about 50) of generations makes this system appropriate for long–term monitoring of populations of distinct bacterial strains in gut ecotopes and other natural biocenoses.

The IS1–fingerprinting system performs equally well both in a BIO–RAD MJ mini Personal Thermal Cycler and in the DNA–Technology Thercyc thermal cycler, which is widely available in Russia. PCR in the Thercyc thermal cycler manufactured in Russia requires the application of mineral oil over the PCR mixture to avoid evaporation. Both the yield of the PCR product and the band patterns obtained are perfectly comparable. The use of different polymerases, * Taq * or * Pfu * or their mixture, also did not influence the result (data not shown). Thus, the kinetic features of the equipment used have no definite bearing on the results, which provides an advantage over existing alternative systems for strain typing, such as BOX–PCR, which are more dependent on the quality of equipment and chemicals. The first commercial PCR thermal cycler, Perkin–Elmer Cetus (which became available in 1989), has provided a similar yield and an identical pattern of PCR products.

**Fig. 3:**
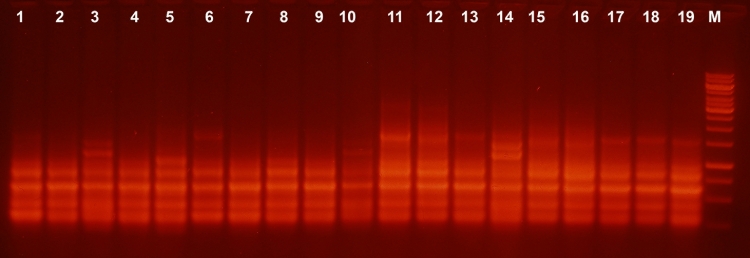
BOX-PCR fingerprinting patterns. Indigenous coliform strains isolated from sample of horse feces No 3 Marker – 1kb DNA ladder (Fermentas)

**Comparison of IS1–fingerprinting, BOX–fingerprinting, and ribotyping of enterobacteria**. We compared the novel method of genomic IS1–fingerprinting with existing methods of molecular BOX–fingerprinting and ribotyping using the same DNA templates as those used for IS1–PCR and complex optimized amplification protocols recommended by the authors of [15]. Electrophoresis of BOX–PCR products in agarose gel demonstrated faint separation of the amplified DNA fragments, whose number averages about 20–30, thus hampering the search for identical patterns without specialized software. The yield of PCR products is lower than that in IS1–PCR. The profiling revealed four identical groups (each combining 2–7 patterns) among the autostrains isolated from the third fecal sample (Fig. 3). Thus, both the discriminative capability and sensitivity of this system are lower than those of the system we offer.

Ribotyping is more labor– and materials–intensive than BOX– and IS1–PCR fingerprinting. This method includes both PCR–amplification of the required DNA sequence and the following enzymatic hydrolysis of the desalted PCR–product. This method did not allow grouping within the given series of field isolates of * E. coli* , thus demonstrating low resolution. This is determined by the highly conserved 16S rRNA gene sequence within the bacterial species and incomplete count of possible mutations in the locus (the endonuclease restriction analysis can only reveal mutations in the restriction site rather than in the entire sequence). The method of choice in this case is DNA sequencing – an expensive and slow process.

**The use of IS1–PCR for differentiation of closely related strains differing in bacteriophage sensitivity**. Susceptibility to infection by distinct phage races is one of the most labile properties of bacteria which rapidly evolve both in natural and laboratory microbial biocenoses. We conducted the following experiment to find out whether the resolution of IS1–fingrprinting is high enough for the differentiation of closely related natural isolates differing in sensitivity to the phages present in the same biocenoses.

Four pairs of autostrains from horse feces were chosen: two with identical IS1 patterns (and identical BOX patterns as well) and two with identical BOX–PCR profiles. These autostrains were tested for sensitivity to a panel of 20 phages differing in specificity, which were preliminarily isolated from horse feces in our laboratory [6]. Phage lysis plaques were only found in isolates with identical IS1 patterns, when coliphages Nos 12 and 17 were applied. At the next step, our own phages were isolated from the same fecal samples from which the enterobacterial strains tested were derived. Fecal extracts were seeded onto the lawns of the tested strains on LB agar according to the bilayer method, and 200 plaques were collected. Each of the autostrains was tested for sensitivity to this phage sampling. The experiment showed that the strains with identical BOX–PCR patterns are differently sensitive to this phage sampling and, interestingly, show different IS1 patterns. In contrast, the strains with identical IS1 patterns are equally sensitive to bacteriophages.

The coliform strains unable to yield the PCR product in our system are relatively rare (5–10% of the tested strains) in samples of highly heterogeneous bacterial associations found in horse feces. In addition, sometimes the strains whose patterns contain few bands cannot be differentiated with adequate certainty.

To increase the number of bands in patterns and thereby increase the resolution of the system, we constructed a series of primers specific to the inverted terminal repeats of other, less distributed in coliform genomes, insertion elements (IS2, IS3, IS4, IS5, and IS30), as well as the primers TR8D, TR8R, and TR8834 complementary to transposase gene sequences presented in many copies in the genomes of many *E. coli* strains.

**Fig. 4:**
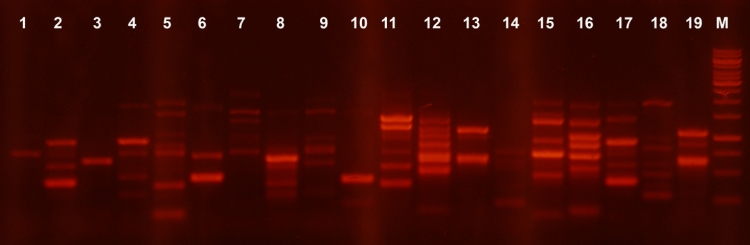
IS1-fingerprinting. The set of the strains and the order are as in Fig. 3 Marker – 1kb DNA ladder (Fermentas)

**Fig. 5:**
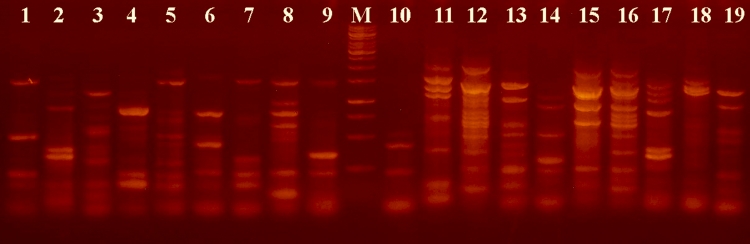
Fingerprinting using the IS1 and TR8834 primer pair. The strains are the same as in Fig. 3 and Fig. 4 Marker – 1kb DNA ladder (Fermentas)

PCR with these primers was carried out as described above. Various oligonucleotide combinations were tested, and the best result was achieved with an IS1 and TR8834 primer pair. The autostrains showing 2–3 electrophoretic bands in IS1–fingerprinting patterns (Fig. 4) might be far easier differentiated after PCR with the IS1+TR8834 primer pair yielding 5–7 bands (Fig. 5). The PCR protocol with this primer combination was the same as that used for IS1–fingerprinting. We compared this improved system with BOX–PCR and ribotyping on the same templates and were convinced of its superiority over these methods.

Therefore, the high resolution of this system can often mask the genetic kindred of distantly related strains. This makes it virtually impossible to classify IS1–fingerprinting profiles into operational taxonomic units (OTUs) or use them as a backbone for phylogenetic tree construction, as is common for other PCR fingerprinting systems, such as ERIC–PCR [9] and BOX–PCR. This reduces the applicability of our system for a series of tasks, such as the search for sanitary–representative enterobacterial strains pointing to a source of fecal pollution [5]. Besides, IS1–profiling focused on coliforms is less universal as relates to the spectrum of analyzed microflora than the above–mentioned systems. Despite our improvements, a small portion of the strains remain nontyped in our method, because of the lack of PCR–amplification.

At the same time, the high resolution of the system we have developed, which is comparable with that of phage typing, makes it the “method of choice” in studies of the microecology of enterobacterial phages in natural microbial ecotopes, such as mammalian gut and wastewater. The environmental conditions of these ecotopes often condones a quick co–evolution of phages and their hosts, leading to unusual heterogeneity of bacterial populations at the strain level [14, 20]. As a result, even closely related strains may essentially differ in sensitivity to bacteriophages inhabiting the ecosystem [6, 8]. It seems obvious that the high resolution of the system, together with its excellent reproducibility, is valuable in many other tasks, in particular, in tracing epidemiological chains in the analysis of the distribution of pathogenic enterobacteria among animals, particularly humans.

## CONCLUSIONS

We have developed a quick system of genomic PCR–fingerprinting that essentially supplements the existing set of tools for molecular differentiation of enterobacteria and enables to resolve the tasks associated with the detailed analysis of highly heterogeneous and rapidly evolving natural populations of these bacteria.

## ABBREVIATIONS

IS: insertion sequence
ERIC: enterobacterial repetitive intergenic consensus
REP: repetitive extragenic palindromic sequence
dNTP: deoxyribonucleotide triphosphate
OTUs: operational taxonomic units
